# The assessment of untreated surface orthodontic mini-implants’ osseointegration through three successive methods

**DOI:** 10.25122/jml-2025-0022

**Published:** 2025-08

**Authors:** Paula Argentina Jiman, Mihaela Felicia Băciuț, Simion Bran, Alexandrina Muntean, Andreea Simona Pop, Cristian Dinu, Lucian Barbu-Tudoran, Alexandru-Flaviu Tăbăran, Romelia Pop, Aranka Ilea, Ondine Patricia Lucaciu, Meda Romana Simu, Sebastian Candrea, Ioana Porumb, Grigore Băciuț

**Affiliations:** 1 Pediatric Dentistry Department, Iuliu Hațieganu University of Medicine and Pharmacy, Cluj-Napoca, Romania; 2 Maxillo-Facial Surgery and Radiology Department, Iuliu Hațieganu University of Medicine and Pharmacy, Cluj-Napoca, Romania; 3 Preventive Dentistry Department, Iuliu Hațieganu University of Medicine and Pharmacy, Cluj-Napoca, Romania; 4 Department of Molecular Biology and Biotechnology, Faculty of Biology, Babeş Bolyai University, Cluj-Napoca, Romania; 5 Department of Pathological Anatomy, Faculty of Veterinary Medicine, University of Agricultural Sciences and Veterinary Medicine, Cluj-Napoca, Romania; 6 Department of Oral Rehabilitation, Iuliu Hațieganu University of Medicine and Pharmacy, Cluj-Napoca, Romania

**Keywords:** mini-implants, skeletal anchorage, osseointegration, orthodontic treatment, removal torque, EDS, histology, MI, Mini-Implant, EDS-EDX, Energy Dispersive X-Ray Spectrometry, MIT, Maximum Insertion Torque, SEM, Scanning Electron Microscopy, Tq, Torque, O, Oxygen, Ca, Calcium, C, Carbon

## Abstract

Mini-implants (MIs) with untreated surfaces are conventionally retained solely through mechanical forces, without any secondary retention mechanism involved (osseointegration). A previously reported issue is the fracture of untreated surface MIs during removal, after orthodontic treatment. Several factors, including potential osseointegration, may cause these fractures. The current research investigates the possibility of osseointegration of untreated surface MIs using three consecutive techniques: removal torque (Tq) measurement using a customized device, immediately followed by spectroscopy analyses (EDX/EDS-Energy Dispersive X-ray Spectrometry and Scanning electron microscopy-SEM), as well as several histological methods to detect the presence of newly-formed bone-cells, which were seen as an indicator for osseointegration. This observational study involved the analysis of removed untreated surface MIs from patients (with a mean age of 21.58 years and a median value of 17 years) at the end of the MI treatment phase. While the EDS, SEM technique, and analysis of removal Tq suggested the presence of osseointegration on the surface of the MIs, the histological methods disproved these results.

## INTRODUCTION

Anchorage in orthodontics is crucial for favorable treatment outcomes. The undesired effects can be minimized through the use of absolute skeletal anchorage. Hence, mini-implants (MIs) can be placed in almost any location in the oral cavity, rendering them very popular among orthodontists for over three decades [1-4]. Furthermore, their advantages outweigh the disadvantages [5,6].

Bone retention of orthodontic MIs is achieved through both mechanical retention (primary stability) and bone remodeling (secondary stability), for treated surface MIs [7]. To increase the resistance of these small-sized MIs, which must withstand high orthodontic forces, a formula of titanium-aluminum-vanadium (Ti-6Al-4V) has been developed, while stainless steel is also still used for MIs [8]. The characteristics of the alloy should ensure that the properties of the MIs are maintained throughout their usage [9-11]. Nevertheless, studies conducted after removal show surface modifications of the MIs due to the deposition of a protein layer [12] and subsequent changes in their electrochemical properties [13]. Orthodontic MI stability can be quantified through mechanical, radiographic, or histological methods (the last two, only when secondary stability is measured) [14]. Primary stability depends on bone quality, dimensions of the MI, and insertion angle. As a measure of primary stability, insertion Tq is commonly used [15-17]. Secondary stability, also known as osseointegration, is defined as the structural and functional connection between living bone and the surface of an implant, without the interposition of soft tissue. One method for evaluating this is by measuring the removal Tq of the orthodontic MI [18-20]. In a previous clinical practice at the private clinic from which this study’s patients were recruited, several cases of MI fracture occurred during removal after orthodontic treatment. We assumed that osseointegration could be the cause of the fracture, despite the current consensus being that no osseointegration occurs on untreated surface MIs.

Several methods have been described for assessing the osseointegration of untreated surface orthodontic MIs [21-24]. In this study, we applied three complementary techniques: (i) removal torque (Tq) as a measure of osseointegration; (ii) surface characterization by energy-dispersive X-ray spectroscopy (EDS) and scanning electron microscopy (SEM); and (iii) histological analysis of interface deposits on explanted MIs, processed using three different histological approaches. The correlation between the histological analysis and the removal torque was established based on the expectation that both a higher number of bone-tissue-specific cells and a higher removal torque value would be observed if osseointegration was present. To measure Tq at removal, we designed a device adapted from dental implantology to fit the smaller heads of MIs. The safe range of insertion Tq values is between 10 and 15 Ncm [25]. Removal torque, as a measure of osseointegration, has values ranging around 8.1 ± 2.9 Ncm for treated MIs and 3.3 ± 1.9 Ncm for untreated ones [26]. The following method was applied: elemental analysis of the explanted MIs' surface, using EDS and SEM, followed by histological analysis. HistoGel, a cytoblock technique, is a relatively recent and innovative method that enables the efficient processing and examination of small tissue samples or individual cells, which are challenging to process and embed using traditional methods [27-30]. The cytospin, also known as a cytocentrifuge, immobilizes cells onto microscope slides through the application of centrifugal force. Subsequently, the cells are fixed and can be further stained for immunofluorescence, immunocytochemistry, or other types of analyses [31].

The aim of our study was to investigate the presence of osseointegration of untreated surface mini-implants using three consecutive techniques: analysis of removal torque using a customized device, immediately followed by EDS and SEM, as well as several histological methods to detect the presence of newly formed bone cells.

## MATERIAL AND METHODS

### Mini-implants (MIs) and alveolar bone sample

The study included a total of 32 Infinitas™ MIs (DB Orthodontics Limited, West Yorkshire, United Kingdom) (31 of the MIs were used as an aid in the orthodontic treatment, and one was unused) with the following characteristics: small head, conical thread, 9.0 mm length, and 2 mm diameter. Infinitas MIs are made from grade 5 titanium alloy (according to ASTM F67, ASTM F136/ISO5832-2). An alveolar bone sample (0.3 x 0.2 x 0.1mm) was obtained through the extraction of a third molar.

### Study participants

We enrolled a total of 31 patients in this study, who required anchorage augmentation with mini-implants between October 2023 and June 2024. The average age of the patients was 21.58 years, with a standard deviation of 9.56. Regarding the gender distribution, the sample consisted of 14 women and 17 men.

The MIs were classified based on the criterion of their placement (localization), either in the maxilla (*n* = 22) or in the mandible (*n* = 9). All patients included in the study were clinically healthy, without systemic diseases or bone metabolism disorders. The patients reported no use of chronic medications, and they read and signed informed consent forms. Underage patients were included in the study with the consent of their legal guardians.

### MI removal procedure using a custom-made Tq device

**Figure 1:**
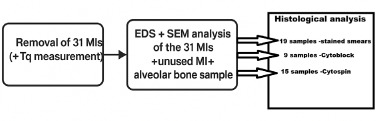
Workflow of the experiment

**Figure 2:**
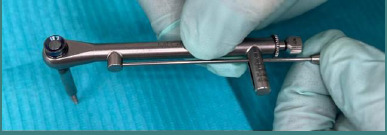
MegaGen Torque Wrench with the adapted device

The first step, as illustrated in Figure 1, involved removing 31 MIs and measuring the removal Tq using our customized device (Figure 2).

**Figure 3:**
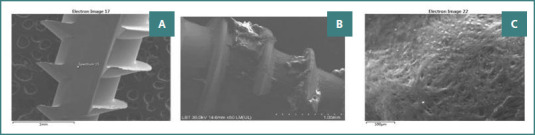
SEM analysis of: A, a new MI; B, an MI after 6 months of bone retention; and C, the alveolar bone.

The retention period was generally between 4 and 14 months. After the anesthesia, the MI was removed using the customized device to measure the removal torque. The removed MIs were collected in hermetically sealed containers to prevent dehydration and contamination.

### EDS and SEM analyses to quantitatively and qualitatively assess deposits on the surface of the MIs

Following removal, each of the 31 MIs was immediately subjected to surface elemental analysis using EDS (Figure 1). Alongside the respective 31 MIs, another unused MI, as well as an alveolar bone sample, were analyzed and compared. SEM was employed to examine the morphology of textures, outlines, and geometric shapes that may be present on the surface of the MIs. EDS Detector Xmas 80 (Oxford Instruments NanoAnalysis, WITec | Raman & Asylum Research, UK) from the National Institute for Research and Development in Isotopic and Molecular Technologies, Cluj-Napoca, Romania, was used for the analyses. We proceeded to the exfoliative collection from the MIs.

### Protocol for Cytoblocks with HistoGel and Paraffin

The removed MIs, along with the Cytobrush-exfoliated surface cells, were suspended in 3 mL of PapSpin solution within 15 mL conical Falcon tubes. After removing the MI and Cytobrush, the solution was centrifuged for 10 minutes at 600x g, and the supernatant was discarded. After fixation with ethanol, the cells were embedded in HistoGel™. The HistoGel block was subsequently embedded in paraffin to create cytoblocks using Epredia™ Citadel 2000 Tissue Processor. These newly obtained paraffin-embedded cytoblocks were sectioned, followed by staining with hematoxylin and eosin.

### Smear preparation and staining protocol

Cells were collected with a Cytobrush from the MI’s surface, then spread on a glass slide and fixed with Diff-stain fixative solution. After drying, the smears were stained using Diff Quik coloration.

### Protocol for the Cytospin technique

The removed MIs, as well as the cells sampled using a cytobrush from the MI’s surface, were suspended in 0.3 ml of PapSpin™ solution within 15 ml conical Falcon tubes. After removing the MI and the cytobrush from the solution, 200 µL was pipetted into the cytofunnel, which was attached to the glass slide. The cell-containing slide was centrifuged using a Cytospin™ 4 Centrifuge (Thermo Scientific). After air-drying, the slides were stained with Diff Quik coloration.

### Histological analysis of the specimens obtained

Three groups of samples were formed based on the histological technique used to prepare them: the Cytoblock technique was employed for nine samples, the Cytospin technique for 15, and the classic stained smear technique for the analysis of 19 samples, resulting in a total of 43 analyzed samples. The number of samples (43) is higher than the number of MIs (31) because some of them were subjected to more than one sample preparation technique for histological analysis.

Each sample was prepared according to the protocols mentioned above. After drying, all slides were labeled with a code for each group and examined microscopically with the assistance of an examiner from the Department of Pathological Anatomy, Faculty of Veterinary Medicine, University of Agricultural Sciences and Veterinary Medicine Cluj-Napoca, Romania. Quantitative and qualitative evaluation was performed using an Olympus BX51 microscope, with bright-field images captured by an Olympus SP350 digital camera and processed with Olympus CellSens software. For each analysis, histological parameters were established to clearly and reproducibly describe the smears.

### Quantification of histological parameters

Cellularity scores were assigned as follows: 1, poorly cellularized; 2, moderately cellularized; and 3, well cellularized. Presence of bacteria: 0 - no bacteria detected, 1 - few bacterial cells, 2 - moderate bacterial cells, and 3 - numerous bacterial cells. Presence of inflammation, quantified on a severity scale: >10% neutrophils (N)- intense inflammatory (YES score) or <10% N - mild inflammatory (NO score).

### Statistical analysis

The data were initially analyzed using descriptive statistics. The correlation between continuous variables was assessed using Pearson’s correlation coefficient. The Mann–Whitney U test was used to compare continuous (ordinal) data between two groups. To compare the distribution of characteristics across more than two groups, the Kruskal–Wallis test was applied. The null hypothesis associated with this test states that the distribution of the parameter is the same across all categories. If the null hypothesis was rejected, the test was followed by Dunn’s post-hoc analysis for multiple pairwise comparisons to identify which groups differed significantly.

The statistical analysis was performed using IBM SPSS Statistics, version 25. Statistical tests were generally conducted using a level of statistical significance of *P* < 0.05 (Sig.).

## RESULTS

The results presented below were obtained using the three successive methods (removal torque, EDS/SEM, and histology methods). For the 31 analyzed MIs, the removal torque values had a mean of 5.71 Ncm (SD = 4.16) with a median value of 4 Ncm.

### EDS and SEM analyses to quantitatively and qualitatively assess deposits on the surface of the mini-implants

**Figure 4:**
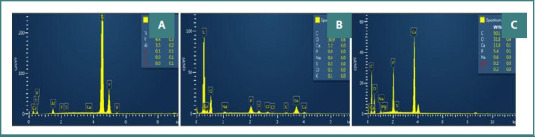
EDS analysis of: A, a new MI; B, an MI after 6 months of bone retention; and C, the alveolar bone.

**Figure 5:**
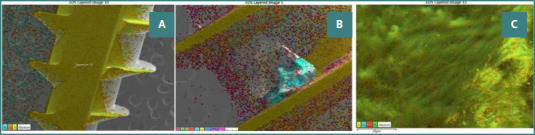
EDS layered analysis of: A, a new MI; B, an MI after 6 months of bone retention; and C, the alveolar bone.

Figures 3, 4, and 5 (A-C) illustrate the first round of SEM, EDS analysis, and EDS layering of a new (unused) MI, an MI after 6 months of bone retention, and the alveolar bone, respectively.

**Figure 6:**
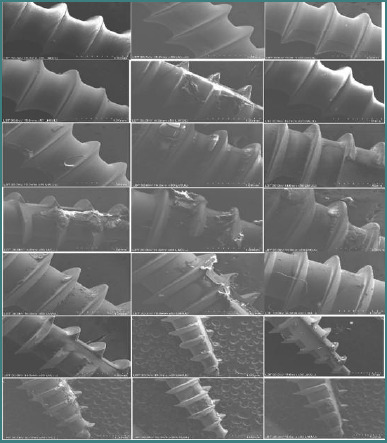
SEM of MIs removed after 4-6 months of retention

The second round of SEM analysis showed that the surface of the unused MIs was free of visible deposits. In contrast, used MIs exhibited deposits of fibroblast-like cells on their surfaces (Figure 6).

**Figure 7:**
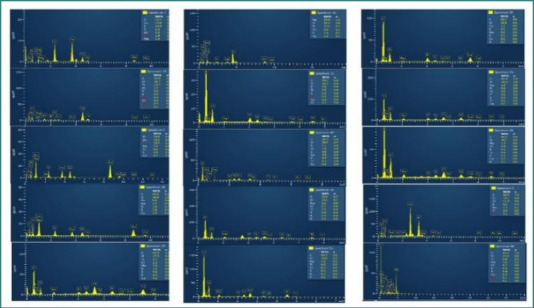
EDS analysis of MIs removed after 4-6 months of retention

**Figure 8:**
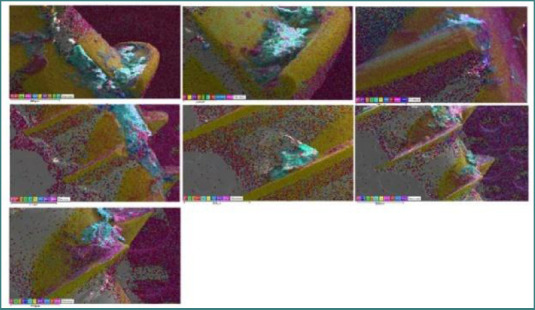
EDS (layered) of removed MIs

The percentages of each element are presented in Figures 4, 7, and 8 based on the EDS spectrum analysis. The percentage composition of carbon (C) for most MIs ranged between 50-60%, of oxygen (O) between 30-40%, and of calcium (Ca) between 1.5-8%. The results are comparable to the elemental composition of the alveolar bone sample. To certify these results, we decided to analyze the layer on the MIs using histological analysis.

### Histological analysis

**Figure 9:**
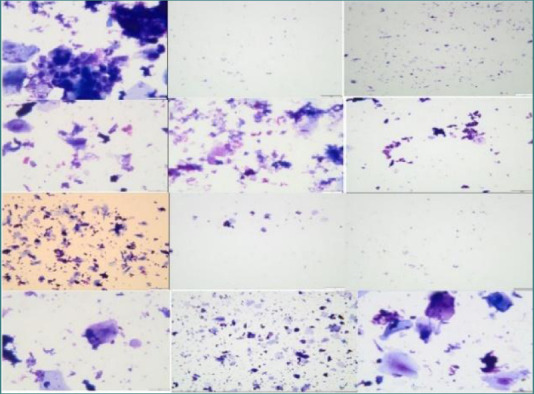
The microscopic appearance of the slides prepared using the cytospin technique

**Figure 10:**
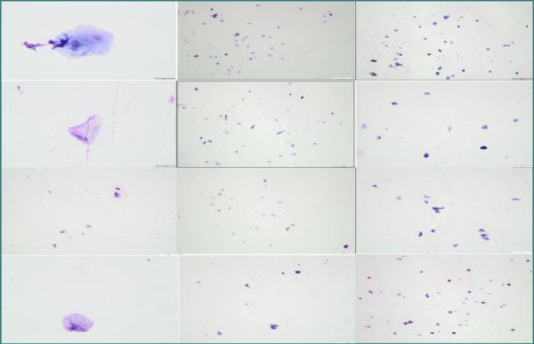
The microscopic appearance of the slides prepared using the stained smear technique

Scores were obtained for the three evaluated parameters, with free bacteria and rarely phagocytosed bacteria (that could be associated with acute/chronic inflammatory processes) being microscopically evident. The bacterial population included cocci, bacilli, and filamentous bacteria. Additionally, a ratio of keratinocytes, neutrophils, and macrophages was observed, along with a rare presence of yeasts, candida-like cells, multinucleated giant cells, and fibroblasts. The microscopic appearance of the slides prepared using the Cytospin technique (Figure 9) revealed the absence of bone cells (e.g., osteoblasts, osteoclasts, osteocytes) and the presence of keratinocytes (50-90%), erythrocytes (30-50%), neutrophils, and bacteria (cocci, bacilli, and in some cases, fusiform bacteria). In the stained smear slides (Figure 10), keratinocyte-like squamous cells were observed, and in some rare cases, bacteria and/or neutrophils were also present. No bone cells (osteoblasts, osteoclasts, osteocytes) were observed.

**Figure 11:**
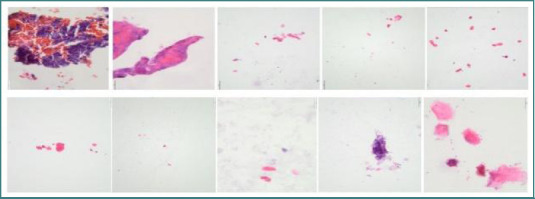
The microscopic appearance of the slides prepared using Cytoblocks with HistoGel and Paraffin

In the preparations using the cytoblock technique (Figure 11), keratinocyte-type cells were observed, and in some rare cases, bacteria, neutrophils, and/or erythrocytes were also present. No bone cells (e.g., osteoblasts, osteoclasts, osteocytes) were present.

### Statistical analysis

The main findings from the statistical analysis are presented below. Referring to removal Tq, Pearson’s correlation coefficient indicated a significant positive correlation between removal torque (Tq) and time of retention (coefficient = 0.516; Sig. = 0.00). Additionally, removal Tq was positively correlated with patient age (coefficient = 0.509; Sig. = 0.00). No significant correlation was found between removal Tq and the other evaluated characteristics (%C, %O, %Ca).

Further comparisons were conducted based on location (maxilla vs. mandible) and gender (women vs. men). According to the Mann-Whitney U test, none of the characteristics—removal Tq (Sig. = 0.71), time of retention (Sig. = 0.45), %C (Sig. =0.31), %O (Sig. = 0.64), and %Ca (Sig.=0.81)—differed significantly between the two localization groups.

Across gender groups, the null hypothesis—that the distribution of removal torque is the same for both male and female categories—was rejected (Sig. = 0.00). Therefore, statistically significant differences were observed in removal torque values between the two groups. Descriptive statistics showed that the mean removal torque was higher in women (7.43 Ncm) compared to men (4.29 Ncm). For the other variables—time (Sig. = 0.06), %C (Sig. = 0.13), %O (Sig. = 0.74), and %Ca (Sig. = 0.13)—the distributions did not differ significantly between the two groups.

The 31 EDS measurements of deposits on mini-implants (Group 1) were compared with those from alveolar bone (Group 2). The comparison of values between the two groups indicates that, at the 0.05 significance level, the differences were not statistically significant, and the null hypothesis was therefore accepted, suggesting that the distribution of %C and %Ca was similar in both groups (Sig.=0.082 for %C and Sig.=0.073 for %Ca).

Focusing on the histological analysis, the Cytospin sample preparation technique yielded the highest average scores for all three evaluated parameters: cellularity (mean score = 2.33), bacterial load (mean score = 2.00), and inflammation (mean score = 0.67). The lowest average values for cellularity and bacterial presence were observed in the Cytoblock group, while the classic stained smears group recorded the lowest inflammation score.

With regard to correlations among histological parameters, there were significant positive correlations between cellularity and bacterial presence, cellularity and inflammation (coefficient = 0.471; Sig. = 0.00), and between bacterial presence and inflammation (coefficient = 0.498; Sig. = 0.00). A negative correlation was observed between time of retention and the inflammation score, which reached statistical significance at the 10% level (coefficient = -0.315; Sig. = 0.090). No significant correlations were identified between cellularity and retention time, or between bacterial load and retention time.

To compare the distribution of histological parameters (cellularity, bacterial presence, and inflammation) across the three groups defined by the histological technique (Cytoblock, Cytospin, and classic stained smears), the non-parametric Kruskal–Wallis test was applied. The null hypothesis—that the distribution of cellularity is the same across all groups—was rejected (statistic test = 9.579; Sig. = 0.00). Dunn’s post-hoc pairwise comparisons revealed significant differences in cellularity between the Cytoblock and Cytospin groups (statistic test = -13.711; Sig. = 0.00), as well as between the classic stained smears and Cytospin groups (test statistic = 9.951; Sig. = 0.01).

Similarly, significant differences in bacterial scores were found across the three groups (statistic test = 22.440; Sig. = 0.00). Specifically, significant differences were noted between the Cytoblock and Cytospin groups (statistic test = -19.444; Sig. = 0.00), and between the classic stained smears and Cytospin groups (statistic test = 16.816; Sig. = 0.00). Regarding inflammation scores, significant differences were also found among the three groups (statistic test= 16.902; Sig. = 0.00), with pairwise comparisons showing statistically significant differences between the Cytoblock and Cytospin groups (statistic test = -11.944; Sig. = 0.00), as well as between the classic stained smears and Cytospin groups (statistic test = 13.202; Sig.= 0.00).

Further details (tabular data) on the statistical results are available from the authors upon request.

## DISCUSSION

The present study aimed to assess the effect of MI on untreated surface behavior during orthodontic treatment, focusing on the objective evaluation of osseointegration. In our study, removal Tq measurements averaged 5.71 ± 4.16. It can be observed that both the mean and the standard deviation were higher than the values reported in other studies (3.3 ± 1.9) [26]. These torque results pointed toward the possibility of osseointegration. In addition, the elemental analysis (EDS) provided values comparable to those of the alveolar bone, suggesting the presence of newly formed bone tissue on the surfaces of the MIs, as in our previous study [32]. Consequently, histological analysis was conducted to clarify these findings further.

Regarding the histological methods used in the study, a fourth method was intended to be used — cryogel slides — following a protocol described earlier [33]. However, due to technical reasons, such as cryotome sectioning at 2 µm and the difficulty in fixing and staining the slides, the method was not successful. Only the other three histological sample preparation techniques mentioned previously were maintained. Upon analyzing the samples obtained with the respective techniques, no bone cells (e.g., osteoblasts, osteoclasts, osteocytes) were observed; only mineralized matrix was observed in a few cases. The statistical analysis pointed to a positive correlation between removal Tq and time and age; however, no significant correlation was found between removal Tq and the other evaluated characteristics (%C, %O, %Ca). No significant correlation was observed between localization and the compared characteristics. Removal Tq had significantly higher median and mean values in women versus men. The statistical results suggest that the fracture of the MIs at removal may have been caused by factors other than the occurrence of osseointegration, including patients' individual bone structure, a high value of the MI insertion torque, and metal fatigue [5,34]. Yet, in all analyzed MIs, no bone tissue-specific cells (e.g., osteoblasts, osteoclasts, osteocytes) were detected through the three histological sample preparation methods, despite the EDS and removal torque methods supporting the osseointegration hypothesis. It is possible that cells were lost from the MIs surface during the EDS analysis; however, this remains a hypothesis that requires further investigation.

## CONCLUSION

The measured removal torque values suggest a possible degree of osseointegration. The EDS analysis of the untreated MIs' surfaces, freshly removed, indicated the possible presence of newly formed bone tissue, based on C-, O-, and Ca percentages very similar to those of an alveolar bone sample used for comparison. However, histological analysis of the deposits revealed no presence of bone-derived cells on the surface of the MIs. Currently, there are very few articles published regarding histological and removal torque measurement methods of untreated surface MIs osseointegration, a topic that needs further investigation.

## Data Availability

Further data is available from the corresponding author upon request.
